# Complete genome sequences of butyrate producing *Anaerostipes hadrus* strains BA1 and GIF7 isolated from the terminal ileum of a healthy lean male

**DOI:** 10.1128/MRA.00701-23

**Published:** 2023-09-29

**Authors:** Adrian Low, Maxim Sheludchenko, Huay Ee Cheng, Xiu Qi Koh, Jonathan Wei Jie Lee

**Affiliations:** 1 Department of Medicine, Yong Loo Lin School of Medicine, National University of Singapore, MD6 Centre for Translational Medicine, Medical Drive, Singapore, Singapore; 2 ASEAN Microbiome Nutrition Centre, National Neuroscience Institute, Jln Tan Tock Seng, Singapore, Singapore; 3 Institute for Health Innovation and Technology (iHealthtech), National University of Singapore, Kent Ridge Crescent, Singapore, Singapore; 4 Department of Medicine, Division of Gastroenterology & Hepatology, National University Hospital, Singapore, Singapore; Rochester Institute of Technology, Rochester, New York, USA

**Keywords:** gut bacteria, whole-genome sequence, anaerobes

## Abstract

*Anaerostipes hadrus* strains BA1 and GIF7 were isolated from a healthy man. The complete genomes’ sizes are 2,946,270 bp (BA1) and 2,907,308 bp (GIF7), with high average nucleotide identity (ANIb = 100%) and alignments ≥96.86% between strains. Conversely, both strains share 97.47% (ANIb) identity and ≤77.36% alignments to *A. hadrus* ATCC 29173^T^.

## ANNOUNCEMENT

Short-chain fatty acids are microbial metabolites produced in the colon through the fermentation of non-digestible carbohydrates, with multiple host benefits (1, 2). *Anaerostipes* of *Bacillota* phylum and *Lachnospiraceae* family are known most prominently for producing butyrate (3, 4). There are currently eight formally classified *Anaerostipes* type species isolated from the guts of homeothermic hosts: *Anaerostipes amylophilus* DSM 108065^T^, *Anaerostipes caccae* DSM 13662^T^, *Anaerostipes hadrus* ATCC 29173^T^, *Anaerostipes hominis* JCM 32275^T^, and *Anaerostipes rhamnosivorans* DSM 26241^T^ isolated from humans (3, 5–9). *A. hadrus* ATCC 29173^T^ (=DSM 3319^T^ and =JCM 17467^T^) was first isolated in 1976 (10). A study of Singapore healthy adults’ gut microbiome revealed a high proportion of novel *A. hadrus* metagenomic assembled genomes (11).

*A. hadrus* strains BA1 and GIF7 were isolated from the terminal ileum washings of a healthy male living in Singapore. Single colonies that grew on brain heart infusion (BHI) agar (Oxoid) were sub-cultured in BHI broth (4 mL) for DNA extraction. Cells were lysed using MP Biomedicals bead-beating tubes on a Fast-Prep 24/5G at 6.0 m/sec for 40 s followed by DNA purification using a Promega Maxwell-16-FFS kit without modifications to the manufacturer’s protocol. Paired-end 150-bp reads were sequenced on an Illumina NovaSeq without modifications to TruSeq Nano DNA library prep kit. Default software parameters were applied unless stated otherwise. Trimmomatic v.0.39 was used to trim and remove adapters from Illumina reads (12), which resulted in 19,972,126 (BA1) and 17,837,076 (GIF7) reads. The DNA library was constructed using a ligation sequencing kit (SQK-LSK110; Oxford Nanopore Technologies), with no size selection, and sequenced on a MinION R9.4.1 flow cell. Total ONT reads for GIF7 = 1,198,535 (3,329 Gbp, N50 = 6.1 kb) and BA1 = 1,090,765 (2,524 Gbp, N50 = 5.5 kb) after basecalling, quality control, and adapter removal with Guppy v.3.2.10 (13). Hybrid-assembled closed genomes were obtained using UniCycler v.0.4.9 (≥522 × short-read and ≥856 × long-read coverages) (14). Genomes were analyzed using the Integrated Microbial Genomes pipeline v.5.1.1 (15) (Table 1). Average nucleotide identity based on the BLAST algorithm (ANIb) was used for pairwise comparison between strains (16). Both genomes showed 99.3% completeness and 2.01% contamination as analyzed using CheckM v1.1.3 (17). Comparative analysis of ANIb revealed identical genomes (ANIb = 100%) for >96% genomic alignment (percentage coverage of each genome by aligned regions) between BA1 and GIF7. In contrast, both strains shared lower ANIb and alignments to ATCC 29173^T^ (Table 1) (18). Both strains have the same seven 16S rRNA genes (1,533 bp each), but all seven genes differ in sequence with an average pairwise identity of 99.4% ± 0.43% (SD). A search of the seven 16S rRNA genes against the NCBI 16S rRNA sequences database using megablast in Geneious v.2023.2.1 confirmed that strains BA1 and GIF7 are closest to ATCC 29173^T^ with 99.6%–99.9% pairwise identities (19). A high proportion (18.9%) of KEGG-annotated protein-coding genes (CDS, 1,402) matched proteins for carbohydrate metabolism, including butyrate, propionate, and inositol phosphate pathways, as annotated using BlastKoala v.3.0 (20).

**TABLE 1:** Genome statistics of *Anaerostipes hadrus* strains GIF7 and BA1 and average nucleotide identity (ANIb) to the type strain

Parameter	GIF7		BA1	
IMG genome ID	259315		259314	
	Number	% of total	Number	% of total
DNA total number of bases	2,907,308	100.00%	2,946,270	100.00%
DNA coding number of bases	2,624,543	90.27%	2,660,646	90.31%
DNA G+C number of bases	1,079,784	37.14%	1,097,216	37.24%
				
DNA scaffolds	1	100.00%	1	100.00%
CRISPR count	4		4	
				
Genes total number	2,864	100.00%	2,922	100.00%
Protein coding genes (CDS)	2,760	96.37%	2,818	96.44%
Regulatory and miscellaneous features	13	0.45%	13	0.44%
RNA genes	91	3.18%	91	3.11%
rRNA genes	21	0.73%	21	0.72%
5S rRNA	7	0.24%	7	0.24%
16S rRNA	7	0.24%	7	0.24%
23S rRNA	7	0.24%	7	0.24%
tRNA genes	66	2.30%	66	2.26%
Other RNA genes	4	0.14%	4	0.14%
Protein coding genes with function prediction	2,293	80.06%	2,322	79.47%
Protein coding genes without function prediction	467	16.31%	496	16.97%
Protein coding genes with enzymes	885	30.90%	889	30.42%
ANIb to ATCC 29173^T^ (GenBank accession number AP028031.1)	97.47% (77.36%)^a^		97.47% (76.33%)^a^	

^a^ The percentage pairwise alignments are shown in parentheses.

## Data Availability

Illumina and Oxford Nanopore reads have been deposited in NCBI under the BioProject number PRJNA999467 and assigned accession numbers SRX21177621 (BA1) and SRX21177622 (GIF7) for Illumina and SRX21334976 (BA1) and SRX21334977 (GIF7) for ONT. THe BA1 genome has been assigned accession numbers CP132968.1 (GenBank) and Ga0509882 (GOLD analysis project; Joint Genome Institute). The GIF7 genome has been assigned accession numbers CP132969.1 (GenBank), and Ga0509881 (GOLD analysis project; Joint Genome Institute).
